# Impact on refusal rates of house visits by Red Cross volunteers in Benin

**DOI:** 10.11604/pamj.supp.2020.35.1.20885

**Published:** 2020-02-06

**Authors:** Melaine Aholoukpe, Robert Davis

**Affiliations:** 1 Health and Care Section, Benin Red Cross, Porto-Novo, Benin; 2 American Red Cross, Nairobi, Kenya

**Keywords:** Refusal rates of house visits, Red Cross volunteers, Benin

## To the editors of the Pan African Medical Journal

The Republic of Benin, and its partners, pursue a two-pronged approach to measles vaccination, both through routine immunization from public and private health facilities, and through periodic mass campaigns, typically targeting 9- to 59-month-olds. During the mass campaigns, social mobilization is done both through conventional mass media approaches (TV, radio, newspapers) and, in selected areas, through house to house social mobilization by Benin Red Cross volunteers.

In Benin, measles control had seen good levels of incidence reduction since the catch-up and follow-up campaigns of 2001, 2003, 2005, 2008, 2011 and 2014. The World Health Organization recommends that countries already engaged in accelerated measles control extend their activities to the problems of rubella and congenital rubella syndrome. It is within this framework that Benin undertook in 2019 a mass campaign against measles and rubella, with the objective of protecting all children from 9 months to 14 years of age against both diseases, with post-campaign introduction of the Measles-Rubella (MR) vaccine into the routine immunization schedule.

There were 5,142,466 children targeted by this campaign. In addition to vaccines, 1.8 million children aged 9 months to 5 years of age were targeted for vitamin A supplementation. The major funders of this campaign were the government of Benin, the GAVI Alliance, the UN Childrens Fund (UNICEF), the World Health Organization, and the American Red Cross.

In its role as an auxiliary to the public authorities, Benin Red Cross (BRC) has in recent years committed itself to social mobilization during measles campaigns, targeting in 2019 the high risk communities in Cotonou (the economic capital of Benin) and communities in Abomey-Calavi, Allada, Djougou, Kpomassé, Porto-Novo, Semé-Podji, So-Ava, Tchaourou, Toffo, and Zé. Starting before the 2019 campaign, which lasted from 6 through 11 March, Red Cross volunteers did house to house social mobilization from 2 through 11 March in the areas listed in the table.

It should be noted that the key to success in convincing the refusals was the strong collaboration of the Benin Red Cross with health actors and local elected representatives in the communities. The latter spared no effort to reassure their community of the relevance of vaccination. This mixing between health actors, politico-administrative authorities and Red Cross actors was due to the daily participation of the BRC in daily wrap-up meetings within the Ministry of Health where joint actions were decided according to the refusals notified (Table 1).

**Table 1:** Initial refusers and acceptors among Benin caregivers, 2019 measles/rubella campaign

Region	Total households visited	Total children in the age range	Total initial refusers	Total final refusers
Abomey-Calavi	22,508	51,172	67	0
Allada	16,982	40,517	357	1
Cotonou	90,769	190,999	450	25
Djougou	21,390	55,641	4	4
Kpomassé	10,436	22,754	28	0
Porto-Novo	43,092	95,392	84	24
Semé-Podji	35,259	83202	26	0
So-Ava	11,619	31,862	112	0
Tchaourou	21,033	51,661	49	49
Toffo	15,123	32,888	16	0
Zé	14,514	33,555	26	9
Totals	299,725	689,643	862	111

Source: Social Mobilization in the Framework of the National Measles/Rubella Vaccination Campaign for Children aged 9 Months to 14 Years, with Vitamin A Supplementation for Children aged 9 Months to 5 Years” (Unpublished report, Benin Red Cross, 2019, in French). NB: in the commune of Djougou, there was one ethnic group which, for religious reasons, was categorically opposed to vaccination despite the intervention of health actors and local elected officials. In Tchaourou, the other outlier, the EPI worker in the locality assured us that the cases of refusals were mastered by the end of the campaign with the help of opinion leaders.”

### Reasons for initial refusals

When caregivers were asked to explain why they did not initially intend to vaccinate their children, the reasons most often cited by initial refusers were (in descending order) fear of side effects, distance to the vaccination site, mother´s other activities, family problem or maternal illness, ignorance of hours and location of sites, illness of the child, inconvenient clinic hours, absence of the child, and long waiting times at the sites. Taken as a whole, 87 percent of the initial 862 refusers became accepters after revisits by Red Cross volunteers, sometimes accompanied by community leaders. This underlines the difference between “soft refusers” and “hard refusers.”

## Conclusion

In Benin, as elsewhere, the historic decline in morbidity and mortality from measles and other childhood diseases has been associated with vaccine hesitancy among a minority of parents [1]. Such reluctance can, at least in some contexts, be overcome by interpersonal communication with trusted members of the community, such as Red Cross volunteers.

## Competing interests

The authors declare no competing interests.
